# The correlation between family relationships and brain activity within the reward circuit in adolescents with Internet gaming disorder

**DOI:** 10.1038/s41598-020-66535-3

**Published:** 2020-06-19

**Authors:** Hyunchan Hwang, Jisun Hong, Sun Mi Kim, Doug Hyun Han

**Affiliations:** Department of Psychiatry, Chung Ang University Hospital, Seoul, South Korea

**Keywords:** Psychology, Health care

## Abstract

Disrupted reward circuits and diminished behavioural control have been suggested as the pathophysiologies of Internet gaming disorder (IGD). Family functioning is thought to play an important role in reward-related control. We hypothesized that adolescents with IGD show disrupted patterns of family relationships, which are associated with brain activity within the reward circuit. 42 adolescents with IGD without comorbidities and 41 healthy controls were assessed for family function and psychological states using the Korean Wechsler Intelligence Scale for Children (K-WISC), Korean version of DuPaul’s attention deficit hyperactivity disorder (ADHD) Rating Scale (K-ARS), Young Internet Addiction Scale (YIAS), Children’s Depression Inventory (CDI), Beck Anxiety Inventory (BAI), and the relationship domain of the Family Environmental Scale (FES-R). Brain activity was assessed via resting-state fMRI. Adolescents with IGD showed increased K-ARS, BAI, and YIAS scores, but decreased FES-R and FES-cohesion subscale scores; YIAS scores were negatively correlated with FES-R scores. Brain connectivity from the cingulate to the striatum was decreased, positively correlated with FES-R scores, and negatively correlated with IGD severity. Adolescents with IGD showed disrupted family relationships, which was associated with the severity of the disorder, and dis-connectivity within the reward circuit.

## Introduction

### Internet Gaming Disorder and the Reward Circuit

Although there are ongoing debates on what constitutes an addiction, a pathology, a syndrome, or an impulse control disorder as well as on over-diagnosis^1^, excessive Internet gaming has now been proposed to be included (warranting further study), as “Internet gaming disorder” (IGD), in Section III of the Diagnostic and Statistical Manual of Mental Disorders (DSM-5)^2^ and as “gaming disorder” (GD), in the International Classification of Diseases (ICD-11)^3^.

Several studies have suggested that the pathophysiology of IGD is associated with a disrupted reward circuit and diminished behavioural control^4–6^. In a meta-analysis on functional imaging studies in patients with IGD, Zheng *et al*.^4^ suggested that reward and executive control circuitry play a critical role in the pathogenesis of IGD. Wang *et al*.^5^ suggested that in patients with IGD, sensitivity in the reward circuit is increased, while the ability to control impulsivity effectively is decreased. Lee *et al*.^6^ reported that subjects in the IGD group had thinner right anterior cingulate (ACC) and right lateral orbitofrontal (OFC) cortices than those in healthy controls. In addition, a thinner right lateral OFC in the IGD group was associated with higher impulsivity.

### Family Functioning and the Reward Circuit

Reward processing may be altered in various psychiatric diseases, including addictive diseases and attention deficit hyperactivity disorder (ADHD)^7,8^. The reward circuit consists of the striatum, which is made up of the lentiform nucleus and the caudate nucleus, and the ventromedial prefrontal cortices including the OFC and ACC^9,10^. Imbalance between the striatum and ventromedial prefrontal cortices has been associated with various psychopathologies^11^. For example, a differential activity pattern within the striatum may depend on the phase of reward processing, such as hypoactivity during reward anticipation and hyperactivity during delivery^12^.

Family cohesion and mother-child interactions such as attachment can play an important role in reward anticipation^13,14^. Child attachment styles have been significantly associated with family cohesion^15^. Kuznetsova^16^ reported that family cohesion can prevent the negative effect of sensitivity to reward on externalizing, while Holz *et al*.^13^ reported that early maternal care may prevent a negative familial effect on psychopathology linked to the reward circuitry, such as in ADHD. Pauli-Pott *et al*.^14^ suggested that good maternal responsiveness and sensitivity could predict the development of reward-related control in children.

### Family Functioning and Internet Gaming Disorder

Family functioning is known as one of the crucial factors that play a role in the aetiology and intervention for the phenomenon of excessive Internet gaming^17^. Many studies have suggested that family functioning such as cohesion might be an important trigger in the aetiology of IGD^18,19^. In a systemic review of family factors in adolescent problematic Internet gaming, Schneider *et al*.^20^ reported that poor parent-child relationships were associated with IGD severity, and that good relationships could thus represent a protective factor in the prevalence of IGD. Chiu *et al*.^21^ found good family functioning to be a protective factor against problematic gaming in Taiwan. Liu *et al*.^22^ employed multi-family group therapy for adolescent with internet addiction (including IGD). Torres-Rodríguez *et al*.^23^ included a family intervention module in their treatment program for IGD, with favourable pilot results. Han *et al*.^24^ used cognitive behavioural therapy (CBT) with enhanced family therapy elements for IGD and showed promising results. González-Bueso *et al*.^25^ reported that IGD groups receiving CBT without parent psychoeducation showed higher drop-out rates during treatment than those receiving CBT with parent psychoeducation.

### Hypothesis

We hypothesized that patients with IGD show disrupted patterns of family relationships, compared to healthy control subjects. In addition, we expected that these patterns of family relationships would be associated with brain activity within the reward circuit in patients with IGD.

## Methods

### Participants

Adolescents with IGD but without other psychiatric comorbidities were recruited from a population of 215 adolescents who visited the Online Clinic and Research Center (OCRC) at Chung Ang University Hospital between January 2015 and December 2018. Of all 215 adolescents with problematic Internet gaming habits, 106 patients with IGD were diagnosed with ADHD and IGD, 15 with ADHD and major depressive disorder (MDD) and IGD, 42 with MDD and IGD, and 10 with IGD and other comorbidities. The number of patients with IGD only (pure IGD) was 42. Because all recruited patients were male, we recruited 41 age-matched male healthy adolescents as control subjects, through advertisements in the outpatient department of Chung Ang University Hospital.

All patients and healthy control subjects who visited the OCRC were assessed with the Structured Clinical Interview of the DSM-5 Clinician Version^26^, a semi structured interview guide for major psychiatric disorders and the diagnostic criteria for IGD were based on the DSM-5^2^. All assessments were done by the authors (DHH, JH), who are certified child and adolescent psychiatrists with over 10 years of clinical experience between them. The exclusion criteria were as follows: 1) history of head trauma and psychiatric or medical diseases, 2) intelligence quotient (IQ) < 70, or 3) claustrophobia.

The research protocol for this study was approved by the institutional review board of Chung Ang University Hospital. All procedures were performed in accordance with the Declaration of Helsinki. Written informed consent was collected from all adolescents and from their parents for their children’s involvement in the study.

### Study procedure and family relationships

All participants (adolescents with IGD and healthy controls) were asked to complete questionnaires regarding demographic data and were administered scales assessing their psychological status, the severity of their disorder, and their family relationships. Psychological status, IQ, ADHD, IGD severity, MDD, and anxiety were measured using the Korean Wechsler Intelligence Scale for Children (K-WISC)^27^, Korean version of DuPaul’s ADHD Rating Scale (K-ARS)^28,29^, Young Internet Addiction Scale (YIAS)^30^, Children’s Depression Inventory (CDI)^31^, and the Beck Anxiety Inventory (BAI)^32^, respectively. Family relationships were assessed using the relationship domain of the Family Environmental Scale (FES-R)^33^ that consists of three subscales: family cohesion, expressiveness, and conflict^18,33^. Family cohesion measures how much support and help family members give to each other (e.g. “Family members really help and support one another”). Expressiveness measures how much family members think they can express their feelings to each other (e.g. “Family members often keep their feelings to themselves”). Conflict measures how much anger is openly expressed within the family (e.g. “We fight a lot in our family”). The relationship domain of the FES measures how individual family members view their family functioning; high scores usually mean that the individual sees their family as well functioning, and that they have low maladjustment levels^33–35^.

### Brain image acquisition and processing

All resting-state magnetic resonance imaging (rs-MRI) data were collected on a 3.0 T Philips Achieva scanner. During Rs-MRI scanning. All adolescent were told to lie down and stay awake with eye closed for 720 seconds until 230 volumes were obtained. Using cushions, participant’s heads were stabilized to prevent head movement. fMRI data were collected axially with an echo-planar imaging (EPI) sequence using the parameters below: TR/TE = 3000/40 ms, 40 slices, 64×64 matrix, 90° flip angle, 230-mm FOV, and 3-mm section thickness without a gap. The first 10 volumes were removed for gradient field stabilization.

Data image preprocessing and processing were prepared using the Data Processing Assistant for Rs-fMRI (DPARSFA toolbox)^36^, which works in Statistical Parametric Mapping (SPM12; http://www.fil.ion.ucl.ac.uk/spm/software/spm12/) and the Rs-fMRI Data Analysis Toolkit (REST)^37^. Brain images were collected in slice acquisition, time differences, realigned, normalized, spatially smoothed with a 6-mm Full-Width Half Maximum (FWHM) kernel, de-trended, and temporally band-pass filtered (0.01–0.08 Hz). Based on the results from realignment processing, subjects who showed excessive head movement (a translation greater than 3 mm or rotation motion greater than 2 degrees in any direction) should be excluded from the analysis. However, we did not find any subjects with excessive head motion.

To acquire brain activity within regions of interest (ROIS), the fractional amplitude of low-frequency fluctuations (fALFF) was extracted using the REST software. During preprocessing of functional data, Fisher-transformed correlation coefficients in each pair of ROIs as well as the fALFF difference between the ROIs was calculated using the CONN-fMRI functional connectivity toolbox (version 15)^38^. Kendall’s coefficient of concordance was converted to z-scores for preparing group analyses. The correlation between FES scores and fALFF was then used to find seed regions which were used as a seed-based functional connectivity (FC) analysis.

A seed-based FC analysis was performed using the seed ROI extracted from the previous step of correlation comparison between FES and fALFF. Pearson’s correlation coefficients were gathered from the averaged blood-oxygenation level dependent (BOLD) seed time course in every voxel. The correlation coefficients were then converted to normally distributed z-scores using Fisher’s z-transform.

### Statistics

Demographic and psychological data were compared between the adolescents with IGD and healthy controls using independent t-tests. Correlations between fALFF maps and FES scores were calculated using the SPM12 software package. fALFF values were compared between the adolescents with IGD and the healthy controls using independent t-tests. FC between the seed and other regions was also compared between adolescents with IGD and healthy controls using independent t-tests. The resulting maps were thresholded to a *p-*value of <0.05, and false discovery rate (FDR) corrections were applied for multiple comparisons with an extent of more than 40 contiguous voxels.

## Results

### Demographic and clinical scale scores

There were no significant differences in age, school education, IQ, and CDI scores between adolescents with IGD and healthy control subjects (Table 1). However, adolescents with IGD showed increased scores on the K-ARS (t = 6.27, p < 0.01), BAI (t = 2.39, p = 0.02), and YIAS (t = 18.58, p < 0.01) and decreased scores on the FES-R (t = −3.73, p < 0.01). Post-hoc tests on the FES-R scores showed that the cohesion subscale scores of the FES-R were lower for adolescents with IGD than for healthy controls (t = −8.76, p < 0.01).

**Table 1:** Comparison of demographic data and clinical characteristics between adolescents with IGD and healthy control subjects.

	Adolescents with IGD	Healthy adolescent	Statistics
Age (years)	14.6 ± 1.1	14.8 ± 2.0	t = −0.67, p = 0.51
School education (years)	7.5 ± 1.0	7.8 ± 1.9	t = −0.92, p = 0.36
IQ	96.4 ± 10.3	96.3 ± 14.0	t = 0.01, p = 0.99
K-ARS	13.6 ± 6.9	5.7 ± 4.3	t = 6.27, p < 0.01*
CDI	7.2 ± 5.2	5.8 ± 3.8	t = 1.40, p = 0.16
BAI	8.1 ± 8.3	4.7 ± 3.4	t = 2.39, p = 0.02*
YIAS	60.6 ± 8.2	30.1 ± 6.6	t = 18.58, p < 0.01*
FES-R	10.5 ± 4.4	14.6 ± 5.4	t = −3.73, p < 0.01*
Conflict subscale	3.5 ± 1.6	4.0 ± 2.7	t = −1.09, p = 0.28
Expression subscale	3.5 ± 1.8	4.2 ± 2.1	t = −1.68, p = 0.10
Cohesion subscale	3.4 ± 1.5	6.4 ± 1.6	t = −8.76, p < 0.01*

K-ARS: Korean version of DuPaul’s ADHD Rating Scale, CDI: Children’s Depression Inventory, BAI: Beck Anxiety Inventory, YIAS: Young Internet Addiction Scale, FES-R: Family Environmental Scale-relationship domain.

All adolescents combined (adolescents with IGD and healthy control subjects) showed a negative correlation between YIAS and FES-R scores (r = −0.50, p < 0.01); within the subgroups, YIAS scores were negatively correlated with FES-R scores in the adolescents with IGD (r = −0.67, p < 0.01) but not in healthy controls (r = −0.11, p = 0.46).

### Correlation between FES scores and fALFF values

**Figure 1:**
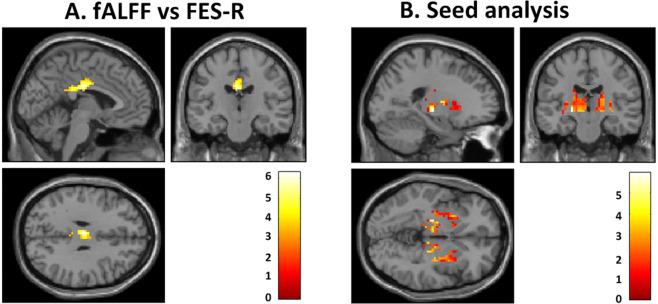
Correlation between brain activity and family relationships and comparison of functional connectivity between adolescents with IGD and healthy control subjects. (**A**) Correlation between Family Environmental Scale-relationship domain (FES-R) scores and fALFF values (fALFF vs FES). Colours indicate correlations between fALFF values within the left cingulate cortex (x, y, z: −3, −18, 30, ke=105, T = 6.30, FDRq=0.002) and FES-R scores in all adolescent (r = 0.66, p < 0.01). (**B**) Comparison of functional connectivity (FC) from the left cingulate seed to other regions between adolescents with Internet gaming disorder (IGD) and healthy control subjects (Seed analysis). The FC from the left cingulate seed to both lentiform nuclei (x, y, z: −21, −18, −3, ke=446, T = 3.96, P_uncorrected_ < 0.001 and ke=394, T = 3.49, P_uncorrected_ < 0.001, 21, −15, 12) was decreased, compared to healthy controls.

In all adolescents combined, the fALFF within the left cingulate cortex (x, y, z: −3, −18, 30, ke=105, T = 6.30, FDRq=0.002) was correlated with the FES-R scores (r = 0.66, p < 0.01) (Fig. 1A). The post-hoc analysis showed a positive correlation between the fALFF value within the left cingulate cortex and the FES-R scores for both the IGD (r = 0.61, p < 0.01) and healthy control groups (r = 0.60, p < 0.01).

### Comparison of FC from left cingulate seed to other regions between adolescents with IGD and healthy controls

The FC from the left cingulate seed to both lentiform nuclei (x, y, z: −21, −18, −3, ke=446, T = 3.96, P_uncorrected_ < 0.001 and ke=394, T = 3.49, P_uncorrected_ < 0.001, 21, −15, 12) was decreased in adolescents with IGD compared to healthy controls (Fig. 1B). There were no regions that showed significant increases in FC in adolescents with IGD compared to healthy controls.

### Correlations between FC values from the left cingulate to the lentiform nuclei

**Figure 2:**
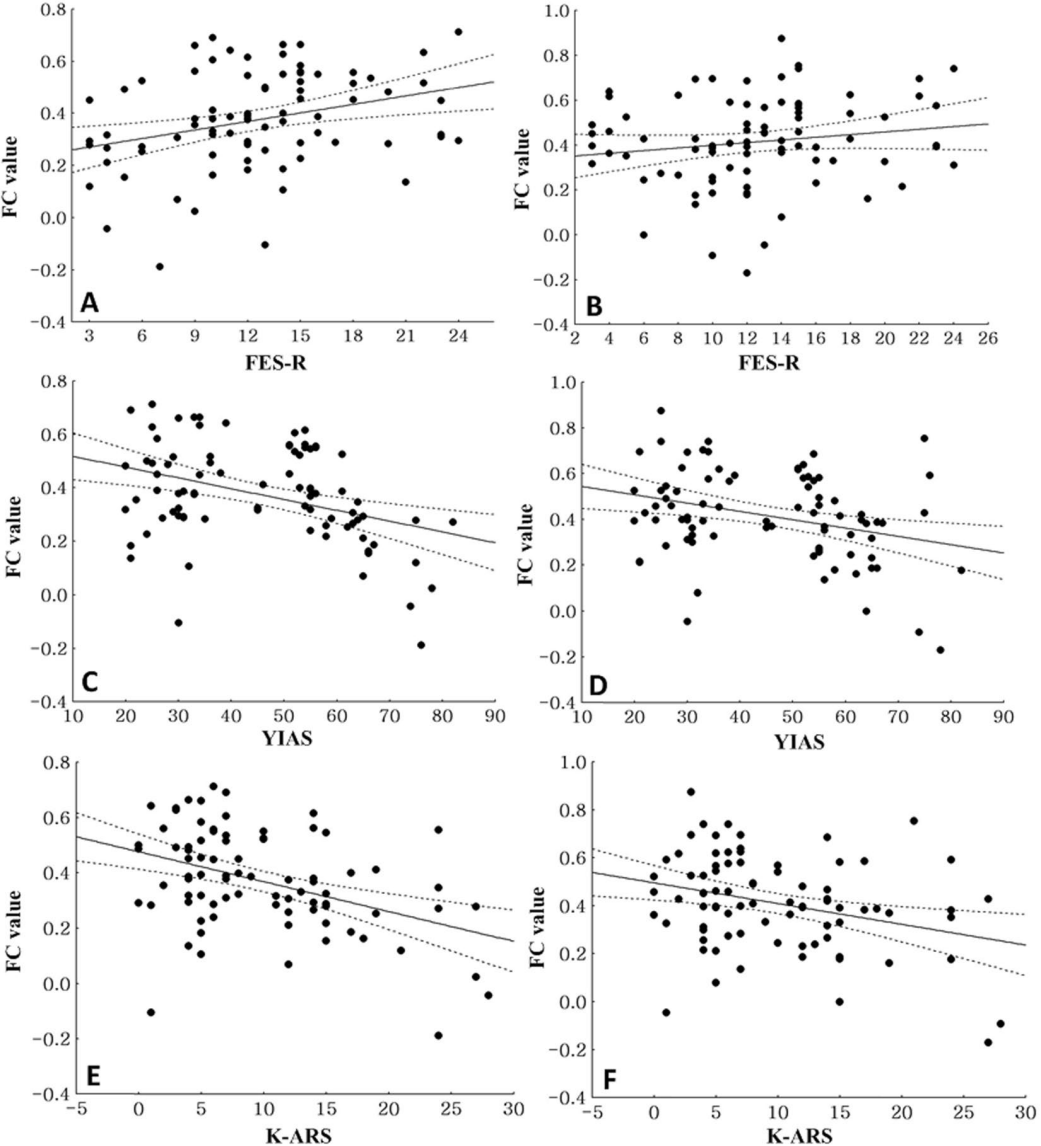
Correlations between FC values from the left cingulate to both lentiform nuclei in all subjects (**A**) Correlation between the functional connectivity (FC) values from the left cingulate to the left lentiform nuclei and the Family Environmental Scale-relationship domain (FES-R) scores in all subjects (r = 0.31, p < 0.01). (**B**) Correlation between the FC values from the left cingulate to the right lentiform nucleus and the Family Environmental Scale-relationship domain (FES-R) scores in all subjects (r = 0.27, p = 0.02). (**C**) Correlation between the FC values from the left cingulate to the left lentiform nucleus and the Young Internet Addiction scale (YIAS) scores in all subjects (r = −0.35, p < 0.01). (**D**) Correlation between the FC values from the left cingulate to the right lentiform nucleus and the Young Internet Addiction scale (YIAS) scores in all subjects (r = −0.37, p < 0.01). (**E**) Correlation between the FC values from the left cingulate to the left lentiform nucleus and the Korean version of DuPaul’s ADHD Rating Scale (K-ARS) scores in all subjects (r = −0.41, p < 0.01). (**F**) Correlation between the FC values from the left cingulate to the right lentiform nucleus and the Korean version of DuPaul’s ADHD Rating Scale (K-ARS) scores in all subjects (r = −0.31, p < 0.01).

In all adolescents combined, the FC value from the left cingulate to the left lentiform nucleus (r = 0.31, p < 0.01) was positively correlated with the FES-R scores. The FC value from the left cingulate to the right lentiform nucleus was also positively correlated with the FES-R scores, but the correlation was not statistically significant (r = 0.27, p = 0.02) (Fig. 2A,B). In all adolescents combined, the FC values from the left cingulate to the left (r = −0.35, p < 0.01) and the right lentiform nucleus (r = −0.37, p < 0.01) were negatively correlated with the YIAS scores (Fig. 2C,D). In all adolescents combined, the FC values from the left cingulate to the left (r = −0.41, p < 0.01) and the right lentiform nucleus (r = −0.31, p < 0.01) were negatively correlated with the K-ARS scores (Fig. 2E,F).

**Figure 3:**
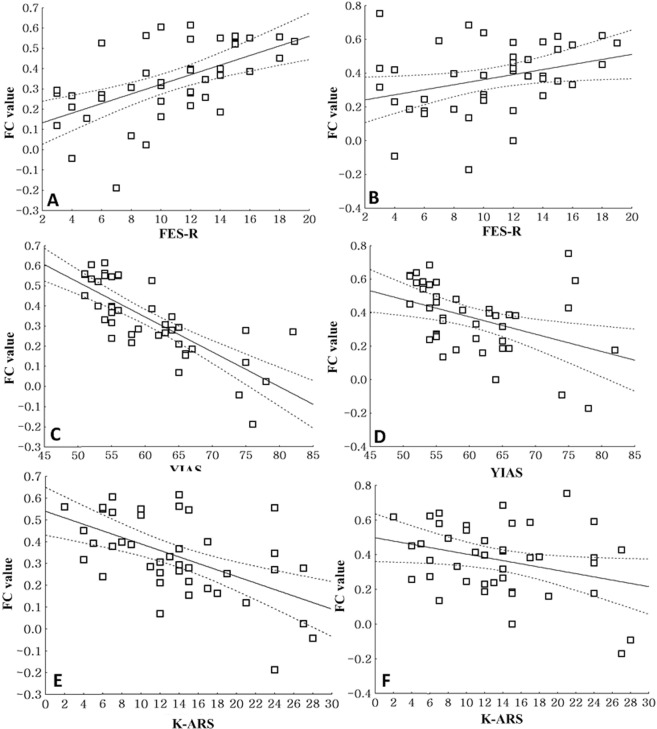
Correlations between FC values from the left cingulate to both lentiform nuclei in adolescents with IGD (**A**) Correlation between the functional connectivity (FC) values from the left cingulate to the left lentiform nucleus and the Family Environmental Scale-relationship domain (FES-R) scores in subjects with internet gaming disorder (IGD) (r = 0.56, p < 0.01). (**B**) Correlation between the FC values from the left cingulate to the right lentiform nucleus and the Family Environmental Scale-relationship domain (FES-R) scores in adolescents with IGD (r = 0.32, p = 0.04). (**C**) Correlation between the FC values from the left cingulate to the left lentiform nucleus and the Young Internet Addiction scale (YIAS) scores in adolescents with IGD (r = −0.67, p < 0.01). (**D**) Correlation between the FC values from the left cingulate to the right lentiform nucleus and the Young Internet Addiction scale (YIAS) scores in adolescents with IGD (r = −0.41, p < 0.01). (**E**) Correlation between the FC values from the left cingulate to the left lentiform nucleus and the Korean version of DuPaul’s ADHD Rating Scale (K-ARS) cores in adolescents with IGD (r = −0.55, p < 0.01). (**F**) Correlation between the FC values from the left cingulate to the right lentiform nucleus and the Korean version of DuPaul’s ADHD Rating Scale (K-ARS) cores in adolescents with IGD (r = −0.31, p < 0.01).

In adolescents with IGD, the FC values from the left cingulate to the left (r = 0.56, p < 0.01) and the right lentiform nucleus (r = 0.32, p = 0.04) were positively correlated with the FES-R scores (Fig. 3A,B), while the FC values from the left cingulate to the left (r = −0.67, p < 0.01) and the right lentiform nucleus (r = −0.41, p < 0.01) were negatively correlated with the YIAS scores (Fig. 3C,D). In adolescents with IGD, the FC values from the left cingulate to the left (r = −0.55, p < 0.01) and the right lentiform nucleus (r = −0.31, p < 0.01) were negatively correlated with the K-ARS scores (Fig. 3E,F).

There were no significant correlations between FES-R scores, YIAS scores, and FC values from the cingulate to both lentiform nuclei in healthy control subjects.

## Discussion

Our results showed increased YIAS scores but decreased FES-R and FES-cohesion scores in adolescents with IGD compared to healthy controls. YIAS scores were negatively correlated with FES-R scores in adolescents with IGD, and brain connectivity from the cingulate to the striatum was decreased. In addition, brain connectivity from the cingulate to the striatum was positively correlated with FES-R scores and negatively correlated with IGD severity in the IGD group.

Adolescents with IGD had higher scores on the K-ARS and BAI than healthy controls, even after excluding adolescents with IGD with other psychiatric comorbidities, implying that adolescents with IGD may have high levels of attention problems and anxiety. Moreover, the FC values from the left cingulate to both lentiform nuclei were negatively correlated with the severity of ADHD scores in all adolescents, including those with IGD. These data are consistent with our previous studies using fMRI to compare patients with ADHD to those with IGD; that study showed a decrease in FC between the right-middle frontal gyrus and the caudate nucleus and between the left cingulate and the caudate nucleus in patients with IGD and those with ADHD, implying that the two groups might share some common pathophysiology^39^. Our earlier EEG study comparing patients with ADHD with comorbid IGD and those with pure ADHD showed a higher relative beta in the comorbid group, suggesting that patients with ADHD, who have difficulty concentrating, might use games as a way to focus their attention^40^. Similar correlations have been found by other researchers regarding attention problems in patients with IGD^41,42^. Regarding anxiety problems in patients with IGD, Wang *et al*.^43^ found that these patients were more likely to have generalized anxiety disorder than healthy controls. Yen *et al*.^44^ showed that patients with IGD used less cognitive reappraisal and more suppression, which in turn resulted in more symptoms of anxiety, compared to healthy control participants.

We found decreased FES-R and FES-cohesion scores in adolescents with IGD. In addition, the FES-R scores were negatively correlated with the YIAS scores in all adolescents combined, whereas only the adolescents with IGD showed the same negative FES-R–YIAS correlation. The relationship dimension of the FES assesses how one might perceive the quality of their family’s relationships^45^. This means that adolescents with IGD perceive their family’s relationship functions to be poor, and that higher problematic gaming patterns and poorer family relationships are connected to each other. Although the design of our current study does not allow causality to be studied, some researchers have hypothesized that this poor perception of family relationship functions may be one of the reasons for adolescents becoming more obsessed with gaming^46^. Studies have estimated that problematic gamers may use games as a way to escape their problems, and poor family relationships might be the reason theses adolescents with IGD feel that they have no other option than to play games^46,47^. Furthermore, our data showed significantly lower cohesion subscale scores in adolescents with IGD than healthy controls. The cohesion subscale within the FES relationship dimension measures the amount of help and support each family member gives to each other^33^. With less cohesion within the family, the individual may feel disconnected from the family and have difficulty getting support from family members in times of crisis, thereby turning to gaming.

In all adolescents combined, the FES-R scores were correlated with the fALFF within the left cingulate cortex. In the seed analysis, the FC from the left cingulate to the left lentiform nucleus was positively correlated with the FES-R scores. In addition, the FC from the left cingulate to both lentiform nuclei was positively correlated with the YIAS scores. In the IGD group, similar results were observed, indicating that lower FC between the cingulate gyrus and the lentiform nuclei was associated with bad family relationships and more severe IGD. Interestingly, the cingulate cortex and the lentiform nuclei are known as part of the reward circuit^9,10^. Moreover, the reward circuit is thought to be linked to family cohesion and attachment^13,14,16^. Our data shows that dysfunctional family relationships relate to dysfunctional reward circuits in the individual, which could be associated with higher IGD symptoms. Earlier studies have suggested that family therapy might have a beneficial effect on IGD^24^.

Our results, which show IGD adolescents have disrupted family relationships and that disruption is correlated with the reward circuit, are in line with prior studies that show child-parent relationships are an important element in IGD^17–21^. To explain the relationship between family relations and IGD, Throuvala *et al*. proposed that poor family relationships could lead to poor self-concept which may result in excessive gaming^48^. A longitudinal study showed that malfunctioning family relationships increased the chance of the child developing problems related to gaming^49^. Another longitudinal study noted similar results in anxious gamers, although high levels of family cohesion after a certain point did not further reduce the risk of IGD, which might indicate there may be more aspects to consider in IGD than just family cohesion^50^. Our study adds new light to this subject, not in causality, but in that we show correlation of IGD and family relationship through a neurobiological point of view. This could be implemented as evidence for family therapy based interventions in IGD. Many family therapy based treatments have already shown efficacy in treating IGD^22,24,25^. A short 3-week family therapy has shown to change game related cues within the brain in IGD patients^51^ and systemic-motivational therapy, a type of narrative family system model used for treatment of substance use disorder, has also been proposed to be helpful when modified for IGD^52^.

The current study has several limitations. First, the sample size was small; hence, the results cannot be generalized. Second, we did not use the entire FES, in order to save time, as adolescents have a tendency to give up or respond erratically and are also prone to social desirability biases when scales get longer^18^. This choice, although improved the overall quality of the scale data, prevented us from including other family-related dimensions, such as personal growth or system maintenance, in the analysis. Third, although the YIAS, which was used as the psychometric assessment scale in our study, is widely used in similar research, it was developed as a measure for general internet addiction and not specifically for IGD. As there have been recent developments in the framework of IGD, initiated by both the American Psychiatric Association and the World Health Organization, future studies could be improved by using scales that incorporate these developments, such as the Internet Gaming Disorder-20 Test^53^, the Internet Gaming Disorder Scale-Short Form^54^, the Internet Gaming Disorder Scale^55^, and the Gaming Disorder Test^56^. Finally, since this was a cross-sectional study, we could not draw clear conclusions on the exact causal relationships between IGD symptoms, dysfunctional reward circuits, and dysfunctional family relationships. Readers should be cautious of interpreting the results of this current study.

In conclusion, adolescents with IGD had disrupted family relationships, which were associated with the severity of the disorder. In addition, disrupted family relationships in adolescents with IGD were associated with dis-connectivity within the reward circuit.

## Data Availability

All data generated or analyzed during this study are included in this published article
